# Cytotoxic T cell recognition of an HIV-1 reverse transcriptase variant peptide incorporating the K103N drug resistance mutation

**DOI:** 10.1186/1742-6405-3-21

**Published:** 2006-09-14

**Authors:** Lisa Mahnke, David Clifford

**Affiliations:** 1 Department of Medicine, Washington University School of Medicine, Saint Louis, USA; 2 Departments of Neurology and Medicine, Washington University School of Medicine, Saint Louis, Missouri, USA

## Abstract

During HIV-1 infection, cytotoxic T cell (CTL) responses exert strong selective pressure on the replicating virus population. Here we report evidence for T cell activity against the drug resistant K103N region of viral reverse transcriptase in three HIV-1 infected patients exposed to NNRTI antiretroviral drugs. We further characterize the response in one patient by ELISPOT analysis. A nine amino acid peptide incorporating 103N was recognized by patient T cells whereas the wild type was not. The RT K103N mutation is selected by the NNRTI class of HIV drugs. We hypothesize that, in certain individuals, CTL responses against 103N-containing epitopes may protect against NNRTI drug resistance. Characterizing such responses in the context of HLA subtypes could lead to tailored HIV drug therapy or to the design of therapeutic vaccines.

## Findings

During HIV-1 infection, anti-viral CTL responses develop and persist [1,2]. Unfortunately, CTL escape mutations are selected, helping the virus evade immune surveillance [3-5]. Almost every region of the HIV proteome can serve as a CTL target, including RT [6]. However, individual class I HLA alleles restrict 8–10 amino acid epitopes such that individuals vary in CTL epitope usage [8].

A few studies have demonstrated CTL responses directed against HIV drug resistance mutations. An HLA-A2-restricted RT epitope including the lamivudine-associated M184V mutation has been characterized [9]. In another study, epitopes incorporating M41L, L74V, M184V and T215Y in RT were demonstrated by ELISPOT [10]. Finally, Karlsson and colleagues have shown that a drug-resistant mutant region in HIV protease can be a CTL target [11]. These studies suggest a dynamic interaction between anti-viral immune responses and the selection of drug resistance mutations during therapy.

The K103N mutation is a common cause of RT NNRTI drug resistance. Clinically, this mutation is potent as a single mutation and causes cross resistance to drugs in this class [12]. We hypothesized that certain individuals can mount T cell responses against epitopes incorporating 103N. If true, then individuals lacking such responses might be predisposed to developing drug resistance at this site.

We recruited 10 patients, 6 men and 4 women, chronically infected with HIV-1 who were exposed to the NNRTI drug efavirenz. The study was approved by the Washington University Human Studies Committee, Study #04-1207. All patients had recent negative serology for hepatitis C and were without fever. Blood samples were drawn after informed consent was obtained. Subjects ranged in age from 30–60 years old with CD4 counts between 334–786 × 10^3 ^cells/ml. 9 had undetectable HIV RNA levels (less than 40 copies/ml) by the Roche Amplicor assay (Roche Diagnostics) within the last 6 months and had no evidence of K103N on a standard HIV genotype (Quest Diagnostics) within the last 5 years. The tenth, subject 2, had a recent viral load of 2,000 copies/ml on a non-NNRTI based regimen and had evidence of circulating K103N by genotype 8 months prior to the study.

A second sample was drawn for subject 6705A 12 months after the initial blood draw. Subject 6705A is a 30 year old African American woman diagnosed with HIV in 2002. She initiated a regimen of lamivudine, AZT and efavirenz in October of 2004, at which time a viral load was 34,600 copies/ml with a CD4 count of 230 × 10^3 ^cells/ml. Within two weeks she developed severe nausea and vomiting. One week later, ectopic pregnancy was discovered requiring surgery and withholding of antiviral medications. She restarted therapy but continued to miss doses. As of December, 2004, and April, 2005, the viral loads remained less than 150 copies/ml, increasing to a range of 1,000–2,000 copies/ml by April and May, 2004. In November 2005, K103N was detected on a standard genotype, at which time the viral load was 5,100 copies/ml.

Peptides were designed based on published sequences [13] and known consensus drug resistance mutations. Peptides were synthesized by Sigma Genosys (The Woodlands, TX) at 96–99% purity as follows: KKKKSVTVL, KKNKSVTVL, HPAGLKKKK, HPAGLKKNK, LGIPHPAGLKKNKSVTVL, GLKKNKSVTVLDVGDAYF, GLKKKKSVTVLDVGDAYF with the underlined residue representing position 103 in RT. The control peptide was AASIRLRPGGKASASA. Stock solutions were made by resuspending peptides in water or acidic solution as recommended by the manufacturer; all peptides were soluble. Peptides were designed using Peptgen [14] for maximal MHC binding prediction.

PBMC were isolated from the peripheral blood on Ficoll-Hypaque (Sigma, St. Louis, MO) by centrifugation. PBMC were plated on IFN-γ-coated 96 well plates (Endogen, Rockford, IL) at a density of 1 × 10^5 ^cells per well using RPMI 1640 media with glutamine supplementation (Sigma, St. Louis, MO) and 10% fetal calf serum. The peptide stocks were diluted in RPMI media and added to the wells at a final concentration of 14 μg/ml each. 5 μg/ml PHA (Sigma, St. Louis, MO) was used as a stimulant control. The plates were incubated 24 hours at 37°C, 5% CO_2 _and developed according to the manufacturer's instructions. Briefly, the plates were washed then incubated with INF-γ detection antibody (Endogen). After washing, plates were incubated with streptavidin-AP conjugate secondary antibody (Endogen). After a final washing, NBT/BCIP substrate (Endogen) was added and the color was developed. Spots were counted manually using a dissection microscope. In the initial screen, wells were considered positive if total counts were greater than 10 spots per well. Recombinant IFN-γ controls produced intense staining; PHA control showed innumerable spots; media-only control produced less than 3 spots per well (data not shown). For subject 6705A, the month 12 blood sample was analyzed by ELISPOT by plating PBMCs 1.3 × 10^5 ^or 5 × 10^4 ^per well in triplicate. HLA class I typing on whole blood or PBMC fraction genomic DNA was performed at the Washington University School of Medicine HLA laboratory using a commercial assay (Invitrogen) using PCR and HLA-specific probe hybridization.

Results of class I typing are as follows [subject) HLA-A, HLA-B, HLA-C]: 4A) A03/25, B18/35, Cw04/12; 52405A) A02/*, B07/15, Cw03/15; 52605) A02/24, B35/40, Cw03/04; 6105) A03/*, B50/57, Cw07/18; 6205A) A11/30, B42/52, Cw12/17; 6205B) A02/24, B07/27, Cw01/07; 6605B) A02/03, B07/44, Cw05/07; 6605C) A02/*, B18/57, Cw06/07; 6705A) A23/33, B15/58, Cw02/03; 2) A03/23, B35/37, Cw02/07 (*represents likely homozygosity as common A02 or A03 alleles but a rare second allele cannot be excluded).

The IFN-γ ELISPOT assay is known to correlate with cytokine flow cytometry-based methods and is a sensitive test for antigen-specific T cell activity [15]. By this method, we found the region around mutation K103N to be reactive to T cells in 3 of the 10 subjects using 18-mer 103N mutant peptides in an initial screen. Subjects 6205A and 6705A had significant responses to a mixture of LGIPHPAGLKKNKSVTVL plus GLKKNKSVTVLDVGDAYF (peptides assayed together). Subject 2 had a significant response to GLKKNKSVTVLDVGDAYF and a response slightly above background for LGIPHPAGLKKNKSVTVL (data not shown).

**Figure 1:**
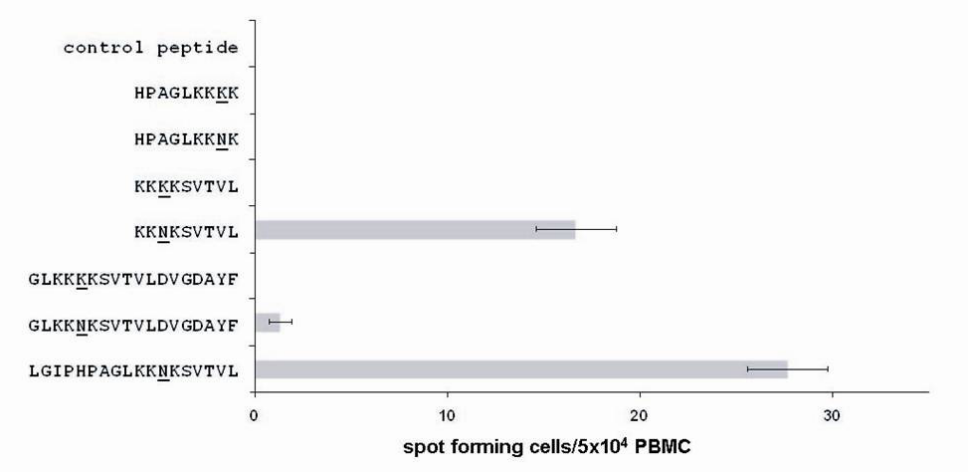
**ELISPOT assay for CTL responses to RT 103 region peptides**. Subject 6705A PBMCs were stimulated with wild type or mutant peptides as described in the text. RT position 103 is underlined. Error bars indicate standard deviations of assays performed in triplicate.

We next verified and characterized an epitope incorporating K103N in subject 6705A using PBMCs obtained 12 months after the initial ELISPOT assay. As shown in Figure 1, individual 18-mer mutant peptides LGIPHPAGLKKNKSVTVL and GLKKNKSVTVLDVGDAYF reacted with the subject's T cells, confirming the previous mixed-peptide result. Wild type peptide GLKKKKSVTVLDVGDAYF did not react. Furthermore, a 9-mer mutant peptide, KKNKSVTVL, reacted significantly whereas the wild type did not (Fig. 1). Finally, neither mutant nor wild type 9-mers HPAGLKK(N/K)K displayed CTL activity (Fig. 1). These results indicate that subject 6705A was able to mount an anti-103N CTL response if 103N was properly positioned within the epitope. The reactivity of such a short peptide suggests that the response is due to CTL activity [8].

If HLA A or B is important for the 103N antiviral response at RT amino acids 101–109 in subject 6705A, then candidate alleles for presentation of this peptide are A23, A33, B15 or B58. Although the alleles were typed only to two digits in this study, among A*2301, A*3301, B*1501 and B*5801, A*2301 can be predicted to have the highest binding affinity for KKNKSVTVL at a predicted IC50 of 613 nM using an *smm *prediction tool [16,17]. It is interesting to note that subject 2 also carries an A23 allele and demonstrated responses against this region.

Because of the relatively long half life of efavirenz, patients who simultaneously discontinue combination antiretroviral therapy are exposed to functional efavirenz monotherapy [18]. Such monotherapy can result in the rapid selection of K103N [19]. For subject 6705A, anti-103N CTL activity may have contributed to suppression of 103N mutant virus replication, as reflected by the relatively low levels of viremia prior to switching drug therapy.

The emergence of K103N and other drug resistant variants may be a dynamic process influenced by patient-specific anti-HIV CTL activity. In support of this idea, interactions between certain HLA subtypes, specific antiretroviral drug exposures and HIV sequence diversity have been found [20]. Another study found CTL responses against drug resistant variants [21] but did not assess the RT 103 region that we describe here. Characterizing more HLA subtypes with regard to HIV epitopes incorporating drug resistance mutations could lead to the design of therapeutic vaccines or suggest tailored therapy against HIV.

## Competing interests

The author(s) declare that they have no competing interests.

## Abbreviations

HIV: human immunodeficiency virus. HLA: human lymphocyte antigen. CTL: cytotoxic T cell lymphocyte. RT: reverse transcriptase. MHC: major histocompatibility complex. NRTI: nucleoside reverse transcriptase inhibitor. ELISPOT: enzyme-linked spot assay. NNRTI: non-nucleoside reverse transcriptase inhibitor. PBMC: peripheral blood mononuclear cells. IFN-γ: interferon gamma. PHA: phytohemagglutinin.

## Authors' contributions

LM conceived of the study, designed the protocol, obtained institutional review board approval, collected and processed samples, performed immunoassays and analyzed the results. DC assisted in study design and data analysis. Both authors read and approved the final manuscript.

## References

[B1] Walker BD, Charkrabarti S, Moss B, Paradis TJ, Flynn T, Durno AG, Blumberg RS, Kaplan JC, Hirsch MS, Schooley RT (1987). HIV-specific cytotoxic T lymphocytes in seropositive individuals. Nature.

[B2] Draenert R, Verrill CL, Tang Y, Allen TM, Wurcel AG, Boczanowski M, Lechner A, Kim AY, Suscovich T, Brown NV, Addo MM, Walker BD (2004). Persistent recognition of autologous virus by high-avidity CD8 T cells in chronic, progressive human immunodeficiency virus type 1 infection. J Virol.

[B3] Allen TM, O'Connor DH, Jing P, Dzuris JL, Mothe BR, Vogel TU, Dunphy E, Liebl ME, Emerson C, Wilson N, Kunstman KJ, Wang X, Allison DB, Hughes AL, Desrosiers RC, Altman JD, Wolinsky SM, Sette A, Watkins DI (2000). Tat-specific cytotoxic T lymphocytes select for SIV escape variants during resolution of primary viraemia. Nature.

[B4] Goulder PJ, Brander C, Tang Y, Tremblay C, Colbert RA, Addo MM, Rosenberg ES, Nguyen T, Allen R, Trocha A, Altfeld M, He S, Bunce M, Funkhouser R, Pelton SI, Burchett SK, McIntosh K, Korber BT, Walker BD (2001). Evolution and transmission of stable CTL escape mutations in HIV. Nature.

[B5] Kelleher AD, Long C, Holmes ED, Allen RL, Wilson J, Conlon C, Workman C, Shanunak S, Olson K, Goulder P, Brander D, Ogg G, Sullivan JS, Dyer W, Jones I, McMichael AJ, Rowland-Jones S, Phillips RE (2001). Clustered mutation in HIV-1 gag are consistently required for escape from HLA-B27-restricted cytotoxic T lymphocyte responses. J Exp Med.

[B6] Walker BD, Flexner C, Paradis TJ, Fuller TC, Hirsch MS, Schooley RT, Moss B (1988). HIV-1 reverse transcriptase is a target for cytotoxic T lymphocytes in infected individuals. Science.

[B7] Altfeld M, Addo MM, Shankarappa R, Lee PK, Allen TM, Yu XG, Rathod A, Harlow J, O'Sullivan K, Johnston MN, Goulder PJ, Mullins JI, Rosenberg ES, Brander C, Korber B, Walker BD (2003). Enhanced detection of human immunodeficiency virus type 1-specific T-cell responses to highly variable regions by using peptides based on autologous virus sequences. J Virol.

[B8] Rudolph MG, Stanfield RL, Wilson IA (2006). How TCRs bind MHCs, peptides, and coreceptors. Ann Review Immunol.

[B9] Schmitt M, Harrer E, Goldwich A, Bauerle M, Graedner I, Kalden JR, Harrer T (2000). Specific recognition of lamivudine-resistant HIV-1 by cytotoxic T lymphocytes. AIDS.

[B10] Samri A, Haas G, duntze J, Bouley JM, Calvez V, Katlama C, Autran B (2000). Immunogenicity of mutations induced by nucleoside reverse transcriptase inhibitors for human immunodeficiency virus type 1-specific cytotoxic T cells. J Virol.

[B11] Karlsson AC, Deeks SG, Barbour JD, Heiken BD, Younger SR, Hoh R, Lane M, Sallberg M, Ortiz GM, Demarest JF, Leigler T, Grant RM, Martin JN, Nixon DF (2003). Dual pressure from antiretroviral therapy and cell-mediated immune response on the human immunodeficiency virus type 1 protease gene. J Virol.

[B12] Clavel F, Hance AJ (2004). HIV drug resistance. NEJM.

[B13] Los Alamos National Laboratory HIV SequenceDatabase. http://hiv-web.lanl.gov/content/hiv-db/mainpage.html.

[B14] Los Alamos HIV Database, HIV Molecular Immunology. http://hiv-web.lanl.gov/content/immunology.

[B15] Karlsson AC, Martin JN, Younger SR, Bredt BM, Epling L, Ronquillo R, Varma A, Deeks SG, McCune JM, Nixon DG, Sinclair E (2003). Comparison of the ELISPOT and cytokine flow cytometry assays for the enumeration of antigen-specific T cells. J Immunol Methods.

[B16] Immune Epitope Database and Analysis Resource. http://www.immuneepitope.org.

[B17] Peters B, Sidney J, Bourne P, Bui HH, Buus S, Doh G, Fleri W, Kronenberg M, Kubo R, Lund O, Nemazee D, Ponomarenko JV, Sathiamurthy M, S choenberger S, Stewart S, Surko P, Way S, Wilson S, Sette A (2005). The immune epitope database and analysis resource: from vision to blueprint. PLoS Biol.

[B18] Ribaudo HJ, Hass DW, Tierney C, Kim RB, Wilkinson GR, Gulick RM, Clifford DB, Marzolini C, Fletcher CV, Tashima KT, Kuritzkes DR, Acosta EP (2006). Pharmacogenetics of plaslma efavirenz exposure after treatement discontinuation: an adult AIDS Clinical Trials Group study. Clin Infect Dis.

[B19] Bacheler LT, Anton ED, Kudish P, Baker D, Bunville J, Krakowski K, Bolling L, Aujay M, Wang XV, Ellis D, Becker MF, Laut AL, George HJ, Spalding DR, Hollis G, Abremski K (2000). Human immunodeficiency virus type 1 mutations selected in patients failing efavirenz combination therapy. Antimicrob Agents Chemother.

[B20] John M, Moore CB, James IR, Mallal SA (2005). Interactive selective pressures of HLA-restricted immune responses and antiretroviral drugs on HIV-1. Antivir Ther.

[B21] Mason RD, Bowmer MI, Howley CM, Gallant M, Myers JCE, Grant MD (2004). Antiretroviral drug resistance mutations sustain or enhance CTL recognition of common HIV-1 Pol epitopes. J Immunol.

